# Multifarious and Interactive Roles of GRAS Transcription Factors During Arbuscular Mycorrhiza Development

**DOI:** 10.3389/fpls.2022.836213

**Published:** 2022-03-28

**Authors:** Tania Ho-Plágaro, José Manuel García-Garrido

**Affiliations:** Department of Soil Microbiology and Symbiotic Systems, Zaidín Experimental Station (EEZ), CSIC, Granada, Spain

**Keywords:** arbuscular mycorrhiza, GRAS transcription factors, transcriptional regulatory network, transcriptional complexes, symbiotic plant genes

## Abstract

Arbuscular mycorrhiza (AM) is a mutualistic symbiotic interaction between plant roots and AM fungi (AMF). This interaction is highly beneficial for plant growth, development and fitness, which has made AM symbiosis the focus of basic and applied research aimed at increasing plant productivity through sustainable agricultural practices. The creation of AM requires host root cells to undergo significant structural and functional modifications. Numerous studies of mycorrhizal plants have shown that extensive transcriptional changes are induced in the host during all stages of colonization. Advances have recently been made in identifying several plant transcription factors (TFs) that play a pivotal role in the transcriptional regulation of AM development, particularly those belonging to the GRAS TF family. There is now sufficient experimental evidence to suggest that GRAS TFs are capable to establish intra and interspecific interactions, forming a transcriptional regulatory complex that controls essential processes in the AM symbiosis. In this minireview, we discuss the integrative role of GRAS TFs in the regulation of the complex genetic re-programming determining AM symbiotic interactions. Particularly, research being done shows the relevance of GRAS TFs in the morphological and developmental changes required for the formation and turnover of arbuscules, the fungal structures where the bidirectional nutrient translocation occurs.

## Introduction

Arbuscular mycorrhiza (AM) is a mutual symbiosis between soil-borne fungi from the phylum Glomeromycotina and the majority of higher plants. This highly beneficial symbiotic interaction substantially boosts plant growth, development and fitness by facilitating growth and reproduction under mineral-stress conditions (Clark and Zeto, 2000). In exchange, AM fungi obtain their carbon from the host plant in the form of plant photosynthates and lipids (Bago et al., 2003; Luginbuehl et al., 2017). This whole process of bidirectional nutrient exchange between plant and fungus is closely linked to and highly dependent on environmental and biological variables (Smith and Read, 2008).

The formation of AM requires the host root cells to undergo significant structural and functional modifications, leading eventually to reciprocal beneficial effects. A combination of genetic, molecular and cellular studies has shown that functional symbiosis appears to occur following a series of plant-controlled checkpoints. During the establishment of the symbiosis, host plant root cells regulate the development and functioning of arbuscules, which are specialized intraradical and highly branched fungal structures, through complex stage-specific transcriptional reprogramming (Pimprikar and Gutjahr, 2018).

Genes involved in signaling, protein metabolism, nutrient transport, secondary metabolite biosynthesis, cell wall modification and lipid metabolism are activated during symbiosis, suggesting that complex transcriptional regulation is required for AM development and functioning. Consequently, a growing number of accumulating transcripts encoding putative transcriptional regulators as well as certain cis-regulatory elements essential for AM-specific gene expression in arbuscule-containing cells have been described (Rubio et al., 2001; Karandashov et al., 2004; Chen et al., 2011; Pimprikar and Gutjahr, 2018).

Genome-wide characterization and expression studies of TF genes activated during AM in *Petunia* (Rich et al., 2017), *Lotus* (Xue et al., 2015), *Medicago* (Hartmann et al., 2019) and tomato (Ho-Plágaro et al., 2019), have revealed that the GRAS gene family is prominent among the AM-inducible TF genes in plants. Moreover, most of these AM-induced GRAS genes belong to the scarecrow-like (A and B), RAD1, and RAM1 subfamilies, which are absent in the whole non-AM host Brassicaceae family (Cenci and Rouard, 2017), suggesting that these GRAS genes play a specific role during mycorrhization. In this minireview, we discuss the integrative roles of GRAS TFs in the regulation of transcriptional changes associated with AM development.

## A Brief Description of GRAS TFs Functions and Interactions

The acronym GRAS is based on the first three members identified in this family: gibberellin-acid insensitive (GAI), repressor of GA1 (RGA), and scarecrow-like (SCL) proteins (Pysh et al., 1999). GRAS TFs play a crucial regulatory role in a diverse range of fundamental plant biology processes such as plant development, gibberellin signaling, stress responses, and symbiotic processes (Tian et al., 2004; Gutjahr et al., 2015). All GRAS proteins are between 360 and 850 amino acids length and share a common conserved GRAS domain in their C-terminal region, consisting of two leucine heptad repeats (LHRs), as well as the motifs VHIID, SAW, and PFYRE (Pysh et al., 1999). These five motifs constitute the GRAS domain (Hirsch et al., 2009). In contrast, the amino (N-) terminal part of GRAS proteins is variable as well as intrinsically disordered (Sun et al., 2011) and can also include other motifs such as the DELLA motif, which is known to modulate DELLA protein interactions with many structurally diverse TFs (Marín-de la Rosa et al., 2014).

Although several genome-wide analyses have been carried out on the GRAS family, and GRAS genes have been characterized in a number of plant species, their classification has not been fully resolved. Based on a panel of eight representative angiosperm species, Cenci and Rouard (2017) identified 29 orthologous groups for the GRAS gene family and they regrouped them into 17 subfamilies whose names were homogenized based on a review of the literature. Interestingly, having found that certain members were missing from some taxonomic groups, they created five new subfamilies which include the RAD1 and RAM1 subfamilies, reported to be involved in mycorrhizal signaling (Park et al., 2015; Xue et al., 2015) and missing from all Brassicales.

Some of the most representative GRAS protein subfamilies act as regulators of GA signaling and root development, which are important processes that occur during AM formation. DELLA proteins, which share the amino acid sequence DELLA in their N-terminal region, repress gibberellin responses (Silverstone et al., 1998). The SCARECROW (SCR) and SHORT-ROOT (SHR) transcription factors are both involved in radial root organization (Cui et al., 2014), while the SCARECROW-LIKE3 (SCL3) transcription factor, which mediates GA-promoted cell elongation during root development, acts as a coordinator of GA/DELLA and SCR/SHR pathways in *Arabidopsis* (Heo et al., 2011; Zhang et al., 2011). Nodulation Signaling Pathway 1 (NSP1) and Nodulation Signaling Pathway 2 (NSP2) GRAS TFs regulate the Nod factor–induced transcriptional responses in legume species (Smit et al., 2005). However, members of these groups also play a role in mycorrhization, acting as positive regulators of strigolactone (SL) biosynthesis in *Medicago truncatula* and *Oryza sativa* (Liu et al., 2011).

In a simple biological model of transcriptional regulation, gene expression regulation is mediated by the action of transcription factors (TFs) which directly bind promoter cis-elements. However, the functioning of TFs is often mediated by their synergistic and combinatorial capacity to interact with other transcription factors and other transcriptional regulators (TRs) to form regulatory complexes (Gutjahr et al., 2015). Many GRAS proteins have been found to be associated with promoter regions. Surprisingly, in some cases, the targeted promoters also correspond to other GRAS genes, or even to the same GRAS genes (reviewed by Bolle, 2016). However, in most of the experiments performed, it is not possible to discern whether a protein is directly bound to DNA or whether it is part of a complex bound to the chromatin. To date, the direct binding of GRAS TFs to DNA has been confirmed for only very few GRAS proteins (Hirsch et al., 2009; Ma et al., 2014; Li et al., 2016). This, together with the involvement of GRAS proteins in so many diverse processes, suggests that most GRAS proteins do not bind directly to DNA and thus act as TRs rather than TFs (Bolle, 2016). Specific interactions of GRAS proteins with many other interactor proteins have been described. In addition, GRAS proteins, even from different subfamilies, have been shown to be able to interact to form heterodimers, which are often necessary for GRAS protein functionality (Bolle, 2016).

## GRAS Interactions and AM Symbiosis Regulation

Initial evidence of the action of GRAS factors in mycorrhization processes emerged from a comparative study between the processes of nodulation in legumes, as well as mycorrhization in most plant species (Hirsch et al., 2009; Liu et al., 2011). The discovery and characterization of the GRAS TF RAM1 (Required for Arbuscular Mycorrhization 1; Park et al., 2015; Rich et al., 2015; Xue et al., 2015; Pimprikar et al., 2016; Müller et al., 2020) and subsequent identification of many other GRAS transcription factors as central regulators of arbuscule development in plants forming arbuscular mycorrhiza (Floss et al., 2013; Yu et al., 2014; Xue et al., 2015; Heck et al., 2016; Hartmann et al., 2019; Ho-Plágaro et al., 2019) point out the relevance of the GRAS family for mycorrhiza development.

Little is known about how combinations of different GRAS protein TFs and TRs control AM formation and functionality. Several studies have identified direct interactions between GRAS proteins during AM, suggesting that networks of GRAS TFs are necessary to regulate mycorrhization and that AM-related GRAS proteins act synergistically and in a combined manner. Interactions between GRAS proteins appear to play a particular role in regulating arbuscule development and here we focus on the integrative role of these GRAS TFs in the regulation of morphological and developmental changes associated to the accommodation and arbuscule functionality in inner cortical cells.

Functional arbuscule development needs host cells to be increased in size to accommodate the AM fungal structures. Some reports showed that fungal colonization induced cellular changes that affected root morphometric parameters (Russo et al., 2019). The accommodation of AM fungal structures in inner cortical cells impacts cortical root cell development although the molecular mechanisms behind these changes were unknown. Root development is positively regulated by GA in *Arabidopsis* where SCL3 proteins have been shown to interact with GA and DELLA signaling through interactions with plant-specific INDETERMINATE DOMAIN (IDD) family proteins which physically bind to both DELLA and the promoter sequence of the *SCL3* gene (Yoshida et al., 2014). Conversely, GA negatively regulate root system development in *M. truncatula* (Fonouni-Farde et al., 2019) and the SHR–SCR module in cortical cells in this legume showed a distinct expression pattern than in *Arabidopsis* (Dong et al., 2021). Interestingly, the SHR–SCR module in *Medicago* is enable to couple cell division with rhizobial infection (Dong et al., 2021).

Curiously, the differential regulation of root developmental processes in *Arabidopsis,* a non-host plant unable to form AM, and legume plants is accompanied by the absence in *Arabidopsis* of several GRAS TFs specific for mycorrhizal development. Then, it is tempting to speculate that GRAS factors from SCL, SHR, and SCR subfamilies, which are implicated in radial root organization and that mediate GA-promoted cell elongation during root development, are also part of the complex system regulating AM development in roots.

In this sense, Ho-Plágaro et al., revealed that, in addition to some classic GRAS transcription factors involved in AM symbiosis, members of the GRAS subfamilies SHR, SCL3, SCR, and SCL32, which form a regulatory module for the root elongation process (Heo et al., 2011; Zhang et al., 2011), are also involved in regulating mycorrhizal processes in tomato and showed specific expression in cells containing arbuscules (Ho-Plágaro et al., 2019). Previous evidence demonstrated that MIG1 (Mycorrhiza Induced GRAS1) is induced in colonized cortical cells and, together with DELLA, promotes cell expansion to accommodate the developing arbuscule (Heck et al., 2016). Recently, Seeman and co-workers characterized two new MIG (MIG2 and MIG3) and one SCL3 GRAS transcription factors that are induced in arbuscule-containing cells and act as positive or negative regulators of cortical cell size. MIG3 interacts with SCL3 in a transcriptional complex to modulate the activity of the central regulator DELLA and antagonizes the positive action of MIG1 and DELLA in cortical cell size (Seeman et al., 2022). It seems clear that the regulation of cell size to accommodate arbuscules in root cortical cells is controlled by a fine-tuned regulated network of interactive GRAS transcription factors from the DELLA, SCL, SHR, and SCR subfamilies. Thus, it is expected that research addressed to this issue will provide new and interesting results in deciphering the complex regulatory circuits coordinating arbuscule formation and root cell morphology.

In addition to its role in rearranging cell morphology to house arbuscules, the action of DELLA is essential for arbuscule development. In a complex containing CYCLOPS and other proteins, DELLA activates *RAM1* transcription (Pimprikar et al., 2016), and consequently the expression of genes involved in arbuscule development. RAM1 target genes include plant carbohydrate and lipid metabolism genes such as RAM2 (encoding a glycerol-3-phosphate acyltransferase), as well as genes encoding membrane proteins which are essential for the formation and functioning of the arbuscules, such as AM-induced phosphate transporter genes (Park et al., 2015; Bravo et al., 2017; Luginbuehl et al., 2017). Furthermore, RAM1 interacts with RAD1 (Xue et al., 2015) and two other *M. truncatula* AM-related GRAS TFs, TF80, and TF124 (Park et al., 2015), supporting the idea that all these regulators interact to control arbuscule development. RAD1 (Required for Arbuscule Development 1) is a GRAS TF closely related to RAM1, and the relative importance of RAM1 and RAD1 in supporting arbuscule development appears to differ between plant species (Park et al., 2015; Xue et al., 2015), pointing to a putative diversification of AM regulatory networks among mycorrhizal host species. Accordingly, transcriptomic analyses of *ram1* mutants from *L. japonicus*, *M. truncatula*, and *P. hybrida*, suggest differences in the RAM1-induced target genes depending on the plant species (Park et al., 2015; Pimprikar et al., 2016; Luginbuehl et al., 2017; Rich et al., 2017).

DELLA has also been shown to be involved in adverse roles during the arbuscule life-cycle. In particular, a transcriptional regulatory complex composed of the GRAS proteins DELLA and NSP1, together with the transcription factor MYB1, a member of the MYB family, forms a regulatory module required for arbuscular degeneration (Floss et al., 2017). While the specific mechanisms of action and regulation remain to be determined, the involvement of DELLA in the modulation of both arbuscule formation and degeneration, seems to depend on the regulatory complexes formed by the differential combination and association of DELLA with specific additional GRAS and other TFs.

Interestingly, downstream targets of RAM1 include genes encoding AP2-domain TFs such as the WRI (WRINKLED) family (MtWRI5a, MtWRI5b, and MtWRI5c) in *M. truncatula* (Jiang et al., 2018) and CBX1 (CTTC-BINDING TRANSCRIPTION FACTOR1; LjWRI1) in *L. japonicus* (Xue et al., 2018). These are members of the APETALA2 TF family that have also been described as differentially regulated upon mycorrhization (Xue et al., 2015; Ho-Plágaro et al., 2019). During AM formation, these AP2/ERF domain transcription factors regulate host genes involved in phosphate uptake and fatty acid biosynthesis. *L. japonicus* CBX1 and *M. truncatula* WRI5 directly bind to CTTC and AW motifs in the promoter sequences of genes involved in phosphate transport and fatty acid biosynthesis (Jiang et al., 2018; Xue et al., 2018). In a genome-wide analysis with several AM-competent plant species and some non-AM plants it was shown that CTTC motifs are very common in AM-related genes (Favre et al., 2014), hence indicating that RAM1 GRAS TF regulate reprogramming of mycorrhizal roots through these downstream target TFs that bind CTTC motifs. Curiously, overexpression of WRI5a in *M. truncatula* activates expression of RAM1 and *MtRam1* and *MtWri5a* gene expression has been shown to be interdependent, while WRI5a and RAM1 regulate each other at the transcriptional level, thus supporting a model in which both TFs form a positive feedback loop to regulate AM symbiosis (Jiang et al., 2018).

Further research is needed to determine whether WRINKLED transcription factor proteins are involved in mycorrhizal gene expression independently, cooperatively, or downstream of RAM1 during AM development. The lack of an AWbox-related cis element in the promoter of RAM1 suggests that WRI5a-mediated regulation of this gene might be indirect. The possibility of GRAS/AP2-ERF heterocomplex formation also needs to be explored.

## Concluding Remarks and Perspectives

**Figure 1:**
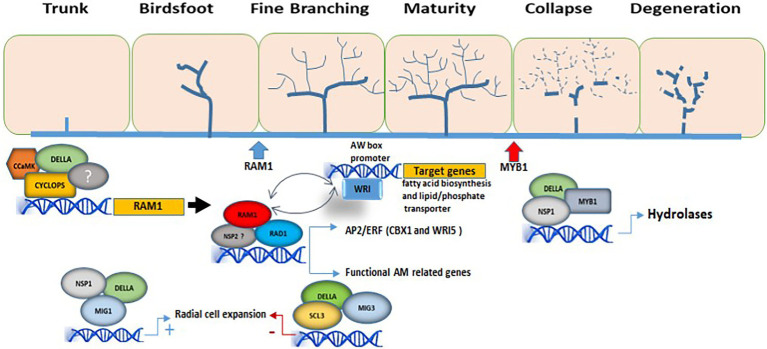
Involvement of GRAS TFs in the regulation of arbuscule formation. The DELLA/CYCLOPS complex regulates the expression of RAM1. In this manner, RAM1 is able to interact with several other GRAS-domain proteins such as RAD1, regulating the expression of genes involved in arbuscule development and functionality, as well as with TFs from the WRI family, activating genes involved in lipid biosynthesis and in nutrient exchanges at the periarbuscular membrane. In this model, WRI and RAM1 regulate each other at the transcriptional level. The interaction of the GRAS-domain protein MIG1 with DELLA and NSP1 is necessary to regulate genes involved in the radial expansion of cortical cells for AM fungal accommodation, while SCL3, together with MIG3 and DELLA, counteracts the positive effect of MIG1 on cell expansion. MYB1 is required for the transcriptional regulation of genes involved in arbuscule degeneration (hydrolytic activity) and interacts with both DELLA proteins and the GRAS-domain protein NSP1. The different stages of arbuscule development are shown. The blue and red arrows mark the beginning of RAM1 activity and MYB1 activity, respectively. Only GRAS TFs with known function during AM are shown.

In this mini review, we discuss the key regulatory role played by GRAS proteins in AM formation, as well as AM symbiotic competence, mainly in arbuscule formation (Figure 1). While their functional significance for symbiosis remains to be further determined, the data suggest the existence of interconnected transcriptional modules that are regulated by multiple GRAS transcription factors. Although the role of GRAS TFs in AM symbiosis appears to be conserved in plants, functional diversification in the GRAS protein repertoire is a basis for variations in AM traits among plant species. Genome-wide characterization and expression studies need to be complemented by protein–protein and protein-DNA interaction studies. Also, further research into the specific inter-GRAS TF interactions and crosstalk, as well as with other TFs and TRs, in addition to identification of regulatory transcriptional modules, would provide a better understanding of how plants are prepared for the establishment of AM symbiosis. AM-forming fungi, whose optimal use would improve plant production in a more sustainable way, are a natural resource that has great potential in agro-biotechnological procedures. Thus, the identification of essential target genes, regulatory modules and downstream processes during AM formation and functioning would be invaluable in order to make AM symbiosis more effective.

## Author Contributions

All authors listed have made a substantial, direct, and intellectual contribution to the work and approved it for publication.

## Funding

This study was supported by grant (PID2020-115336GB-I00) funded by Spanish MCIN/AEI/10.13039/501100011033 and by “ERDF A way of making Europe,” by the “European Union”.

## Conflict of Interest

The authors declare that the research was conducted in the absence of any commercial or financial relationships that could be construed as a potential conflict of interest.

## Publisher’s Note

All claims expressed in this article are solely those of the authors and do not necessarily represent those of their affiliated organizations, or those of the publisher, the editors and the reviewers. Any product that may be evaluated in this article, or claim that may be made by its manufacturer, is not guaranteed or endorsed by the publisher.

## References

[ref1] Bago B. Pfeffer P. E. Abubaker J. Jun J. Allen J. W. Brouillette J.. (2003). Carbon export from arbuscular mycorrhizal roots involves the translocation of carbohydrate as well as lipid. Plant Physiol. 131, 1496–1507. doi: 10.1104/pp.102.007765, PMID: 12644699 PMC166909

[ref2] Bolle C. (2016). “Functional aspects of GRAS family proteins,” in Plant Transcription Factors, Evolutionary, Structural, and Functional Aspects. ed. D. H. Gonzalez (Cambridge: Elsevier), 295–311.

[ref3] Bravo A. Brands M. Wewer V. Dörmann P. Harrison M. J. (2017). Arbuscular mycorrhiza-specific enzymes FatM and RAM2 fine-tune lipid biosynthesis to promote development of arbuscular mycorrhiza. New Phytol. 214, 1631–1645. doi: 10.1111/nph.14533, PMID: 28380681

[ref4] Cenci A. Rouard M. (2017). Evolutionary analyses of GRAS transcription factors in angiosperms. Front. Plant Sci. 8:273. doi: 10.3389/fpls.2017.00273 28303145 PMC5332381

[ref5] Chen A. Gu M. Sun S. Zhu L. Hong S. Xu G. (2011). Identification of two conserved cis-acting elements, MYCS and P1BS, involved in the regulation of mycorrhiza-activated phosphate transporters in eudicot species. New Phytol. 189, 1157–1169. doi: 10.1111/j.1469-8137.2010.03556.x, PMID: 21106037

[ref6] Clark R. Á. Zeto S. (2000). Mineral acquisition by arbuscular mycorrhizal plants. J. Plant Nutr. 23, 867–902. doi: 10.1080/01904160009382068

[ref7] Cui H. Kong D. Liu X. Hao Y. (2014). SCARECROW, SCR-LIKE 23 and SHORT-ROOT control bundle sheath cell fate and function in *Arabidopsis thaliana*. Plant J. 78, 319–327. doi: 10.1111/tpj.12470, PMID: 24517883

[ref8] Dong W. Zhu Y. Chang H. Wang C. Yang J. Shi J.. (2021). An SHR-SCR module specifies legume cortical cell fate to enable nodulation. Nature 589, 586–590. doi: 10.1038/s41586-020-3016-z, PMID: 33299183

[ref9] Favre P. Bapaume L. Bossolini E. Delorenzi M. Falquet L. Reinhardt D. (2014). A novel bioinformatics pipeline to discover genes related to arbuscular mycorrhizal symbiosis based on their evolutionary conservation pattern among higher plants. BMC Plant Biol. 14:333. doi: 10.1186/s12870-014-0333-0, PMID: 25465219 PMC4274732

[ref10] Floss D. S. Gomez S. K. Park H.-J. Maclean A. M. Müller L. M. Bhattarai K. K.. (2017). A transcriptional program for arbuscule degeneration during AM symbiosis is regulated by MYB1. Curr. Biol. 27, 1206–1212. doi: 10.1016/j.cub.2017.03.003, PMID: 28392110

[ref11] Floss D. S. Levy J. G. Lévesque-Tremblay V. Pumplin N. Harrison M. J. (2013). DELLA proteins regulate arbuscule formation in arbuscular mycorrhizal symbiosis. Proc. Natl. Acad. Sci. 110, E5025–E5034. doi: 10.1073/pnas.1308973110 24297892 PMC3870710

[ref12] Fonouni-Farde C. Miassod A. Laffont C. Morin H. Bendahmane A. Diet A.. (2019). Gibberellins negatively regulate the development of *Medicago truncatula* root system. Sci. Rep. 9:2335. doi: 10.1038/s41598-019-38876-1, PMID: 30787350 PMC6382856

[ref13] Gutjahr C. Sawers R. J. Marti G. Andrés-Hernández L. Yang S.-Y. Casieri L.. (2015). Transcriptome diversity among rice root types during asymbiosis and interaction with arbuscular mycorrhizal fungi. Proc. Natl. Acad. Sci. 112, 6754–6759. doi: 10.1073/pnas.1504142112, PMID: 25947154 PMC4450400

[ref14] Hartmann R. M. Schaepe S. Nübel D. Petersen A. C. Bertolini M. Vasilev J.. (2019). Insights into the complex role of GRAS transcription factors in the arbuscular mycorrhiza symbiosis. Sci. Rep. 9, 1–15. doi: 10.1038/s41598-019-40214-4 30833646 PMC6399340

[ref15] Heck C. Kuhn H. Heidt S. Walter S. Rieger N. Requena N. (2016). Symbiotic fungi control plant root cortex development through the novel GRAS transcription factor MIG1. Curr. Biol. 26, 2770–2778. doi: 10.1016/j.cub.2016.07.059, PMID: 27641773

[ref16] Heo J. O. Chang K. S. Kim I. A. Lee M. H. Lee S. A. Song S. K.. (2011). Funneling of gibberellin signaling by the GRAS transcription regulator scarecrow-like 3 in the Arabidopsis root. Proc. Natl. Acad. Sci. U. S. A. 108, 2166–2171. doi: 10.1073/pnas.1012215108, PMID: 21245304 PMC3033297

[ref17] Hirsch S. Kim J. Munoz A. Heckmann A. B. Downie J. A. Oldroyd G. E. (2009). GRAS proteins form a DNA binding complex to induce gene expression during nodulation signaling in Medicago truncatula. Plant Cell 21, 545–557. doi: 10.1105/tpc.108.064501, PMID: 19252081 PMC2660633

[ref18] Ho-Plágaro T. Molinero-Rosales N. Flores D. F. Díaz M. V. García-Garrido J. M. (2019). Identification and expression analysis of GRAS transcription factor genes involved in the control of arbuscular mycorrhizal development in tomato. Front. Plant Sci. 10:268. doi: 10.3389/fpls.2019.00268, PMID: 30930915 PMC6429219

[ref19] Jiang Y. Xie Q. Wang W. Yang J. Zhang X. Yu N.. (2018). Medicago AP2-domain transcription factor WRI5a is a master regulator of lipid biosynthesis and transfer during mycorrhizal symbiosis. Mol. Plant 11, 1344–1359. doi: 10.1016/j.molp.2018.09.006, PMID: 30292683

[ref20] Karandashov V. Nagy R. Wegmüller S. Amrhein N. Bucher M. (2004). Evolutionary conservation of a phosphate transporter in the arbuscular mycorrhizal symbiosis. Proc. Natl. Acad. Sci. U. S. A. 101, 6285–6290. doi: 10.1073/pnas.0306074101, PMID: 15075387 PMC395961

[ref21] Li S. Zhao Y. Zhao Z. Wu X. Sun L. Liu Q.. (2016). Crystal structure of the GRAS domain of SCARECROW-LIKE7 in *Oryza sativa*. Plant Cell 28, 1025–1034. doi: 10.1105/tpc.16.00018, PMID: 27081181 PMC4904676

[ref22] Liu W. Kohlen W. Lillo A. Op Den Camp R. Ivanov S. Hartog M.. (2011). Strigolactone biosynthesis in *Medicago truncatula* and rice requires the symbiotic GRAS-type transcription factors *NSP1* and *NSP2*. Plant Cell 23, 3853–3865. doi: 10.1105/tpc.111.089771, PMID: 22039214 PMC3229154

[ref23] Luginbuehl L. H. Menard G. N. Kurup S. Van Erp H. Radhakrishnan G. V. Breakspear A.. (2017). Fatty acids in arbuscular mycorrhizal fungi are synthesized by the host plant. Science 356, 1175–1178. doi: 10.1126/science.aan0081 28596311

[ref24] Ma Z. Hu X. Cai W. Huang W. Zhou X. Luo Q.. (2014). Arabidopsis miR171-targeted scarecrow-like proteins bind to GT cis-elements and mediate gibberellin-regulated chlorophyll biosynthesis under light conditions. PLoS Genet. 10:e1004519. doi: 10.1371/journal.pgen.1004519, PMID: 25101599 PMC4125095

[ref25] Marín-de la Rosa N. Sotillo B. Miskolczi P. Gibbs D. J. Vicente J. Carbonero P.. (2014). Large-Scale Identification of Gibberellin-Related Transcription Factors Defines Group VII ETHYLENE RESPONSE FACTORS as Functional DELLA Partners. Plant Physiol. 166, 1022–1032. doi: 10.1104/pp.114.244723 25118255 PMC4213073

[ref26] Müller L. M. Campos-Soriano L. Levesque-Tremblay V. Bravo A. Daniels D. A. Pathak S.. (2020). Constitutive overexpression of RAM1 leads to an increase in arbuscule density in Brachypodium distachyon. Plant Physiol. 184, 1263–1272. doi: 10.1104/pp.20.00997, PMID: 32873628 PMC7608154

[ref27] Park H.-J. Floss D. S. Levesque-Tremblay V. Bravo A. Harrison M. J. (2015). Hyphal Branching during Arbuscule Development Requires RAM1. Plant Physiol 169, 2774–2788. doi: 10.1104/pp.15.01155 26511916 PMC4677905

[ref28] Pimprikar P. Carbonnel S. Paries M. Katzer K. Klingl V. Bohmer M. J.. (2016). A CCaMK-CYCLOPS-DELLA complex activates transcription of *RAM1* to regulate arbuscule branching. Curr. Biol. 26, 987–998. doi: 10.1016/j.cub.2016.01.069, PMID: 27020747

[ref29] Pimprikar P. Gutjahr C. (2018). Transcriptional regulation of arbuscular mycorrhiza development. Plant Cell Physiol. 59, 673–690. doi: 10.1093/pcp/pcy024, PMID: 29425360

[ref30] Pysh L. D. Wysocka-Diller J. W. Camilleri C. Bouchez D. Benfey P. N. (1999). The GRAS gene family in Arabidopsis: sequence characterization and basic expression analysis of the SCARECROW-LIKE genes. Plant J. 18, 111–119. doi: 10.1046/j.1365-313X.1999.00431.x, PMID: 10341448

[ref31] Rich M. K. Courty P. E. Roux C. Reinhardt D. (2017). Role of the GRAS transcription factor *ATA/RAM1* in the transcriptional reprogramming of arbuscular mycorrhiza in *Petunia hybrida*. BMC Genomics 18:589. doi: 10.1186/s12864-017-3988-8, PMID: 28789611 PMC5549340

[ref32] Rich M. K. Schorderet M. Bapaume L. Falquet L. Morel P. Vandenbussche M.. (2015). The petunia GRAS transcription factor *ATA/RAM1* regulates symbiotic gene expression and fungal morphogenesis in arbuscular mycorrhiza. Plant Physiol. 168, 788–797. doi: 10.1104/pp.15.00310, PMID: 25971550 PMC4741351

[ref33] Rubio V. Linhares F. Solano R. Martín A. C. Iglesias J. Leyva A.. (2001). A conserved MYB transcription factor involved in phosphate starvation signaling both in vascular plants and in unicellular algae. Genes Dev. 15, 2122–2133. doi: 10.1101/gad.204401, PMID: 11511543 PMC312755

[ref34] Russo G. Carotenuto G. Fiorilli V. Volpe V. Chiapello M. Van Damme D.. (2019). Ectopic activation of cortical cell division during the accommodation of arbuscular mycorrhizal fungi. New Phytol. 221, 1036–1048. doi: 10.1111/nph.15398, PMID: 30152051

[ref35] Seemann C. Heck C. Voß S. Schmoll J. Enderle E. Schwarz D.. (2022). Root cortex development is fine-tuned by the interplay of MIGs, SCL3 and DELLA during arbuscular mycorrhizal symbiosis. New Phytol. 233, 948–965. doi: 10.1111/nph.17823, PMID: 34693526

[ref36] Silverstone A. L. Ciampaglio C. N. Sun T.-P. (1998). The Arabidopsis RGA gene encodes a transcriptional regulator repressing the gibberellin signal transduction pathway. Plant Cell 10, 155–169. doi: 10.1105/tpc.10.2.155, PMID: 9490740 PMC143987

[ref37] Smit P. Raedts J. Portyanko V. Debellé F. Gough C. Bisseling T.. (2005). NSP1 of the GRAS protein family is essential for rhizobial nod factor-induced transcription. Science 308, 1789–1791. doi: 10.1126/science.1111025, PMID: 15961669

[ref38] Smith S. Read D. (2008). “Mycorrhizal Symbiosis. 3”. (Academic Press. London).

[ref39] Sun X. Xue B. Jones W. T. Rikkerink E. Dunker A. K. Uversky V. N. (2011). A functionally required unfoldome from the plant kingdom: intrinsically disordered N-terminal domains of GRAS proteins are involved in molecular recognition during plant development. Plant Mol. Biol. 77, 205–223. doi: 10.1007/s11103-011-9803-z, PMID: 21732203

[ref40] Tian C. Wan P. Sun S. Li J. Chen M. (2004). Genome-wide analysis of the GRAS gene family in rice and *Arabidopsis*. Plant Mol. Biol. 54, 519–532. doi: 10.1023/B:PLAN.0000038256.89809.57, PMID: 15316287

[ref41] Xue L. Cui H. Buer B. Vijayakumar V. Delaux P.-M. Junkermann S.. (2015). Network of GRAS transcription factors involved in the control of arbuscule development in *Lotus japonicus*. Plant Physiol. 167, 854–871. doi: 10.1104/pp.114.255430, PMID: 25560877 PMC4348782

[ref42] Xue L. Klinnawee L. Zhou Y. Saridis G. Vijayakumar V. Brands M.. (2018). AP2 transcription factor CBX1 with a specific function in symbiotic exchange of nutrients in mycorrhizal Lotus japonicus. Proc. Natl. Acad. Sci. 115, E9239–E9246. doi: 10.1073/pnas.1812275115 30209216 PMC6166803

[ref43] Yoshida H. Hirano K. Sato T. Mitsuda N. Nomoto M. Maeo K.. (2014). DELLA protein functions as a transcriptional activator through the DNA binding of the indeterminate domain family proteins. Proc. Natl. Acad. Sci. 111, 7861–7866. doi: 10.1073/pnas.1321669111, PMID: 24821766 PMC4040565

[ref44] Yu N. Luo D. Zhang X. Liu J. Wang W. Jin Y.. (2014). A DELLA protein complex controls the arbuscular mycorrhizal symbiosis in plants. Cell Res. 24, 130–133. doi: 10.1038/cr.2013.167, PMID: 24343576 PMC3879709

[ref45] Zhang Z. L. Ogawa M. Fleet C. M. Zentella R. Hu J. Heo J. O.. (2011). Scarecrow-like 3 promotes gibberellin signaling by antagonizing master growth repressor DELLA in Arabidopsis. Proc. Natl. Acad. Sci. U. S. A. 108, 2160–2165. doi: 10.1073/pnas.1012232108, PMID: 21245327 PMC3033277

